# Fingerprint changes in CSF composition associated with different aetiologies in human neonatal hydrocephalus: glial proteins associated with cell damage and loss

**DOI:** 10.1186/2045-8118-10-34

**Published:** 2013-12-18

**Authors:** Irum Naureen, Khawaja A Irfan Waheed, Ahsen W Rathore, Suresh Victor, Conor Mallucci, John R Goodden, Shahid N Chohan, Jaleel A Miyan

**Affiliations:** 1 Faculty of Life Sciences, The University of Manchester, AV Hill Building, Oxford Road, Manchester M13 9PT, UK; 2 Department of Neonatology, The Children’s Hospital and Institute of Child Health, Ferozepur Road, Lahore, Pakistan; 3 Institute of Human Development, Manchester Academic Health Sciences Centre, The University of Manchester, Oxford Road, Manchester M13 9WL, UK; 4 Neurosurgical Unit, Alder Hey Children’s Hospital, Eaton Road, Liverpool L12 2AP, UK; 5 Department of Neurosurgery, Leeds General Infirmary, Great George Street, Leeds LS1 3EX, UK; 6 Department of Biosciences, COMSATS Institute of Information and Technology, Islamabad, Pakistan

**Keywords:** Hydrocephalus, Human neonates, CSF, GFAP, Vimentin, CNPase, MBP

## Abstract

**Background:**

In hydrocephalus an imbalance between production and absorption of cerebrospinal fluid (CSF) results in fluid accumulation, compression and stretching of the brain parenchyma. In addition, changes in CSF composition have a profound influence on the development and function of the brain and together, these can result in severe life-long neurological deficits. Brain damage or degenerative conditions can result in release of proteins expressed predominantly in neurons, astroglia, or oligodendroglia into the brain interstitial fluid, CSF and blood. Determination of such products in the CSF might be of value in diagnosing cause, aetiology and/or assessing the severity of the neurological damage in patients with hydrocephalus. We therefore analysed CSF from human neonates with hydrocephalus for these proteins to provide an insight into the pathophysiology associated with different aetiologies.

**Methods:**

CSF was collected during routine lumbar puncture or ventricular tap. Samples were categorized according to age of onset of hydrocephalus and presumed cause (fetal-onset, late-onset, post-haemorrhagic or spina bifida with hydrocephalus). Glial fibrillary acidic protein (GFAP), myelin basic protein (MBP), vimentin and 2^′^ , 3^′^-cyclic nucleotide 3^′^-phosphodiesterase (CNPase) were analysed through Western blotting of hydrocephalic CSF samples (n = 17) and compared with data from CSF of normal infants without neurological deficits (n = 8).

**Results:**

GFAP was significantly raised only in CSF from post-haemorrhagic hydrocephalus while MBP was significantly raised in post-haemorrhagic and in spina bifida with hydrocephalus infants. Vimentin protein was only detected in some CSF samples from infants with late-onset hydrocephalus but not from other conditions. Surprisingly, CNPase was found in all neonatal CSF samples, including normal and hydrocephalic groups, although it was reduced in infants with late onset hydrocephalus compared with normal and other hydrocephalic groups.

**Conclusions:**

Apart from CNPase, which is an enzyme, the markers investigated are intracellular intermediate filaments and would be present in CSF only if the cells are compromised and the proteins released. Raised GFAP observed in post-haemorrhagic hydrocephalus must reflect damage to astrocytes and ependyma. Raised MBP in post-haemorrhagic and spina bifida with hydrocephalus indicates damage to oligodendrocytes and myelin. Vimentin protein detected in some of the late-onset hydrocephalic samples indicates damage to glial and other progenitors and suggests this condition affects periventricular regions. The presence of CNPase in all CSF samples was unexpected and indicates a possible novel role for this enzyme in brain development/myelination. Less CNPase in some cases of late-onset hydrocephalus could therefore indicate changes in myelination in these infants. This study demonstrates differential glial damage and loss in the developing human neonatal hydrocephalic brain associated with different aetiologies.

## Background

Hydrocephalus is a pathological condition characterised by an abnormal production and/or absorption of cerebrospinal fluid (CSF). The aetiology of hydrocephalus is unclear, even with some evidence of genetic predisposition [1]. With a global incidence of 1:500 (NIH, USA), hydrocephalus affects between 1:100–1:5000 live human births with a lower incidence in developed countries achieved through elective terminations. Hydrocephalus can result in deficient/abnormal cerebral cortex development and lifelong neurological deficits [2-4]. The pathophysiology of hydrocephalus in neonates remains poorly understood since they do not experience raised intracranial pressure despite accumulation of CSF in the brain accompanied by ventricular and cranial expansion [5,6]. The extent of ventricular dilation depends on the location of CSF blockage and length of time of the block [7,8]. Fetal-onset ventriculomegally (enlarged ventricles without raised pressure) is thought to result in more severe brain damage [3,4] but recent studies challenge this notion [9] even though ventricular expansion, and consequential compression and stretching of the brain must have a damaging effect in severe cases. Currently the only control measure is birth termination and the only treatment option is surgical fluid diversion. Although surgery does address the life-threatening raised intracranial pressure, compression and stretching of the brain parenchyma, it does not recover lost or abnormal development, cellular damage or the life-long neurological issues resulting from these [4-6].

Improvements in prenatal diagnosis and treatment will raise the quality of life for children with hydrocephalus through decreased mortality and morbidity, but this may only be through a better understanding of the underlying aetiology and biological basis of the condition and the development of additional treatments to shunt surgery. The literature in both human and experimental animal studies is not clear with controversy on cause and with much work targeted at understanding the consequences of raised pressure rather than possible earlier events, due in large part to the view of CSF as a mechanical support fluid rather than a fluid with physiological importance. The latter role of CSF is currently under considerable review and renewed research [10-13] with some new insights emerging into the unique part played by CSF in development and brain function [9,14,15]. Recent research has focused on the interplay between CSF signals and their role in normal neurodevelopment [11-13], and there is growing evidence that secreted proteins or other factors within the CSF have an age-dependent effect on neural cell precursor proliferation and cortical development [14]. Thus, major alterations in CSF protein composition in neonatal neurological disease states could have direct and significant effects on global neurodevelopment [16]. Alternatively an imbalance in the levels of specific CSF proteins may directly affect on-going neurodevelopmental processes, which could lead to the developmental defects associated with post-haemorrhagic hydrocephalus [17] as well as those already associated with CSF composition changes in fetal-onset hydrocephalus [9,18,19].

The severity of hydrocephalus is usually assessed from clinical signs and symptoms. In very young patients an increase in head circumference usually occurs with only minor symptoms due to the compliance of the immature skull. CT-scanning and intracranial pressure-monitoring have proved to be valuable tools in the assessment of the diagnosis, but many patients still do not show satisfactory outcomes after CSF shunt surgery [20] suggesting an underlying pathophysiological process may not have been addressed by the procedure. The outcome, in terms of possible neurological deficits, may reflect these underlying processes that are not addressed by shunting and thus do not reflect the brain damage that is directly inflicted by hydrocephalus [4,20,21]. These deficits may not be rescued or recovered [22] and may be a consequence of poor, or abnormal development of the cerebral cortex [18,19]. Thus, although appropriate diagnostics have been developed for hydrocephalus, the potential of biological markers to characterise the underlying pathological processes has so far been overlooked.

The composition of the CSF should reflect physiological/biochemical changes happening in the brain parenchyma, and more particularly in the periventricular white matter and sub-ventricular zones lining the ventricles [23,24] where atrophy of the white matter, and to some extent the grey matter and damage to axons were found in various hydrocephalic conditions [3,4,25-28]. In the neonatal period a major phase of gliogenesis occurs in the ventricular and sub-ventricular zones and it is likely that there is a significant effect from hydrocephalus on developing glial cells, but less effect on the post-mitotic neurones produced prenatally [29-32]. The aim of this study was to test whether different aetiologies of neonatal hydrocephalus were correlated with different profiles of proteins associated with glial damage in human neonatal hydrocephalus.

## Materials and methods

### Patient samples

Prior to the study, ethical approval was obtained from the UK NHS REC (ref: 08/H1015/43), Central Manchester NHS Foundation Trust, Alder Hey Children’s Hospital, Leeds Teaching Hospitals NHS Trust, The University of Manchester, and The Children’s Hospital Lahore. Written informed consent for participation and demographic data including sex, age and ethnicity were obtained from each patient’s parents using their home language with verbal explanation where necessary. This study is based on 25 neonates, 16 males and 9 females varying in age from 1 to 44 weeks (Table 1). CSF samples were collected by trained clinical staff either by lumbar puncture from normal infants (n = 8), presenting with mild fever and suspected meningitis but sera negative, or through insertion of catheters into the brain lateral ventricle to drain fluid from infants with hydrocephalus. All CSF samples were sera negative for known infective organisms. Any sera positive samples were excluded from this study as were all samples with obvious blood contamination. The procedures and tests were part of the routine clinical management of the patients and we obtained fluid excess to clinical test requirements. Samples were frozen at -80°C within 30 min of collection. UK samples were transported frozen on dry ice while samples collected from Lahore Children’s Hospital, Pakistan, were lyophilised (freeze dried) prior to shipment for analysis in the UK. Lyophilised samples were reconstituted to the original volume using deionised water (Milli-Q) before analysis. For obvious clinical reasons it was not possible to obtain CSF samples from the same site in normal and hydrocephalus infants. Total protein would be higher in lumber CSF but other parameters would be expected to be within normal ranges unless damage, inflammation or infection affected specific regions. Any significant differences between hydrocephalus groups were directly comparable to each other as they were all ventricular samples, but any differences to normal lumbar CSF samples needed to be interpreted with due regard to reported differences between lumbar and ventricular CSF. Obstruction of CSF outflow may also affect composition but one aim of the current study is to determine if CSF composition is indeed altered, by whatever mechanism, in hydrocephalus.

**Table 1:** Clinical variables of the normal and hydrocephalic neonates used in the study

**Patient groups**		**Age (days)**			**Sex ratio (male: female)**	**Site of CSF collection**	**Source (UK: Pakistan)**
**Groups**	**n**	**Age range (days after birth)**	**Mean**	**SEM**			
**Normal**	8	8-92	24.50	9.72	5:3	Lumber	0:8
**FOH**	4	11-30	18.25	4.40	4:0	Lateral ventricle	1:3
**LOH**	4	60-300	153.75	55.58	3:1	Lateral ventricle	0:4
**PHH**	5	28-132	74.80	22.05	2:3	Lateral ventricle	5:0
**SB/HC**	4	5-105	41.00	22.79	2:2	Lateral ventricle	4:0

Number of patients, age (days post-partum), gender, and national distribution of the patients studied grouped by known aetiology. Late onset hydrocephalus (LOH) and spina bifida with hydrocephalus (SB/HC) infants are somewhat older than the other groups due to the nature of the condition and the time to receiving treatment. All CSF samples were collected from the lateral ventricle except normal samples that were collected by lumbar puncture. Although there is an age range in each group the low number of samples did not allow analysis of age-related changes. FOH: fetal-onset hydrocephalus, PHH: post-haemorrhagic hydrocephalus.

We received limited information concerning each sample so that sub-categorisation based on severity of hydrocephalus, neurological deficits etc. was not possible in this initial study. Thus, we categorised hydrocephalic CSF samples according to age of onset and the cause of hydrocephalus into the following groups: 1. Fetal-onset or congenital hydrocephalus (FOH) in infants born with classically enlarged heads and associated neurological symptoms (n = 4); 2. Late-onset hydrocephalus (LOH) in infants developing hydrocephalus some days or weeks after birth due to undetermined factors but probably infections (n = 4); 3. Post-haemorrhagic hydrocephalus (PHH) in infants due to an intraventricular or subarachnoid bleed before, during or soon after birth (n = 5); 4. Spina bifida with hydrocephalus (SB/HC) in infants with a primary neural tube defect (n = 4). 5. Control/normal infants with no neurological condition and sera negative for meningitis (n = 8). Sample details are summarised in Table 1.

### Western blotting

All CSF samples were compared through analysis of equal volumes. This was the most meaningful approach since we expected to see changes in total protein as well as in individual components. The specifications for each antibody on the manufacturer’s web sites also listed previous publications characterising the antibody as well as its specificity and utility for identifying the target protein. All samples were diluted 1:1 by volume in Laemmli sample buffer (BioRad 161–0737, Hemel Hempstead, UK) containing mercaptoethanol and heated for 7 min at 95°C. Protein standards were included in each gel as well as a molecular weight standard (Biorad161-0375). Rat (postnatal day 1) astrocyte cell lysates (5–10 μg, see below) were used as a positive control for GFAP and vimentin antibodies. The CNPase antibody was well characterised by previous studies of oligodendrocytes and cortical white matter [33,34]. Furthermore, we had previous data from mass spectrometry that CNPase was indeed present in CSF samples [35]. 5 μl of each CSF sample were separated together with control proteins and molecular weight markers on a 4-12% SDS-PAGE gel and transferred to a PVDF membrane using an iBlot system with blotting kits (Invitrogen, Glasgow, UK). The membranes were blocked using 5% skimmed-milk (Marvel) or 5% fish skin gelatine (Sigma Poole, UK) in 0.1% phosphate-buffer solution (pH 7.4) with 0.1% Tween (Sigma) for 1 hour at room temperature (RT). Primary antibodies were optimised to the following dilutions for 1:4000 (0.25 μg/ml) for rabbit anti-human GFAP (Abcam 7779, Cambridge, UK ), 1:1000 (0.1 μg/ml) for rabbit anti-human Vimentin (Cell Signaling-3932, New England Biolabs, Hitchin, UK), 1:10,000 (0.1 μg/ml) for rabbit anti-human MBP (Thermo Fisher PA1-18324 Lutterworth, UK), 1:1000 (0.1 μg/ml) for rabbit anti-human CNPase (Sigma C9743) were diluted in blocking solution and membranes incubated overnight at 4°C without agitation. Membranes were then washed and incubated in a 1:3000 dilution of the secondary antibody, horseradish peroxidise-conjugated anti-rabbit IgG (Cell signalling 7074) in blocking solution for 1h at RT with agitation, washed and the signals detected using an enhanced chemiluminescence substrate (Amersham Hyper film ECL, GE Healthcare Little Chalfont, UK) and exposed to film.

### Astrocyte cell lysates

In order to produce astrocyte cell lysates for positive control, 1-day old Sprague–Dawley rat pups (Charles River, Ormiston, Scotland) were killed with an overdose of sodium pentobarbitone given by intraperitoneal injection and then decapitated. 2–4 cerebral hemispheres were dissected under sterile conditions in a laminar flow hood. The cortical hemispheres were placed in ice-cold Dulbecco’s modified medium (DMEM, Invitrogen, Glasgow, UK) and further dissected under a stereo microscope (Leica M3Z). The meninges were removed and the choroid plexus removed from the ventricle. All cleaned hemispheres were then digested with trypsin and gently dissociated through decreasing bore sizes of sterile pipettes. The suspension was centrifuged for 10 min at 1000 rpm, the supernatant discarded and the pellet resuspended in DMEM. After a further centrifugation and resuspension to wash the cells, the pellet was resuspended in 10 ml of DMEM per hemisphere. 10 ml of cell suspension was loaded into poly-D-lysine coated 75 cm^2^ flasks and placed in a humidified incubator for 7–9 days at 37°C with 5% CO_2_. After 7–9 day incubation, flasks were sealed tight and shaken on an orbital shaker at 230 rpm for 4 hours at 37°C to separate microglial cells from astrocytes. The media was replaced with 10 ml of fresh DMEM and shaken for a further 1 h. The culture was then washed twice with 5 ml of warm PBS with gentle mixing and then the adherent astrocytes lysed using CelLytic M Cell Lysis Reagent (Sigma) for 15 min at room temperature. Cells were scraped off the flask and the whole media centrifuged to remove cell debris. Protein concentration was measured using a Bradford Assay and 5-10 μg total protein loaded on gels as a control for GFAP and vimentin.

### Densitometry and statistical analysis

Films were scanned and the relative density of bands quantified using Image J (Version 1.44p, NIH). Where no bands were obvious a measure of the staining (presumably background) where the band would have been was used for those CSF samples. Data are presented as mean ± SEM. Results of the experiments were entered into Graphpad Prism V software and the data analysed using one way analysis of variance (ANOVA) and post hoc (Tukey) testing to identify significant differences between groups. The value *p* < 0.05 was considered significant. No distribution tests were carried out and the data was assumed to fall within a normal distribution.

## Results

### Glial fibrillary acidic protein (GFAP)

**Figure 1:**
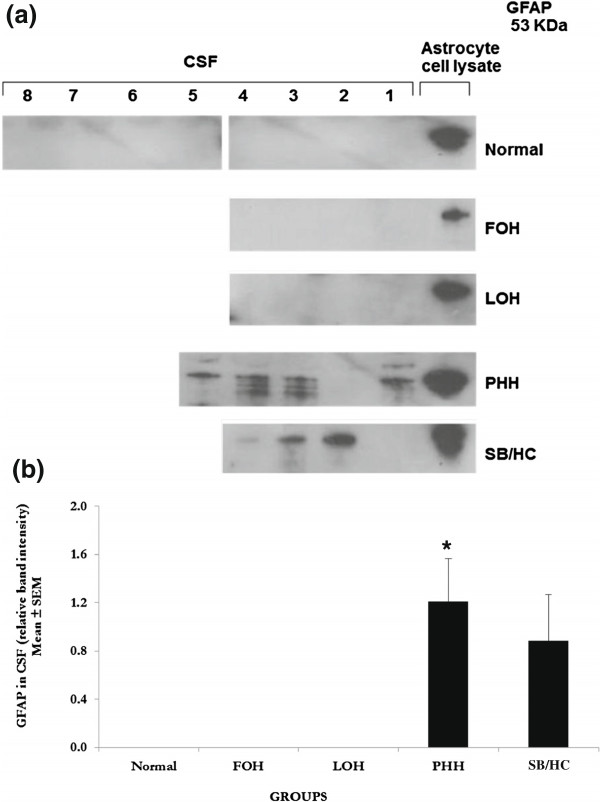
**Western blot analyses for glial fibrillary acidic protein (GFAP). a**. **Western blots for GFAP**. Ten-lane precast gels were used. Each lane had an individual 5 μl sample of CSF from the categories of patients shown. After protein separation using SDS PAGE they were blotted onto a PVDF membrane for probing with antibodies. ECL detection was used to visualise the labelled bands and for semi-quantitative analysis using Image-J software. A single 53 KDa band was labelled using the antibody to GFAP and only found in PHH and SB/HC samples as shown in this representative gel. All samples were run on at least 3 gels with a total number in each group as noted for the semi-quantitative analysis. FOH: fetal-onset hydrocephalus, LOH: late-onset hydrocephalus, PHH: post-haemorrhagic hydrocephalus, SB/HC: spina bifida with hydrocephalus. **b. Semi-quantitative analysis** of the 53 KDa band labelled with anti-GFAP antibody. Values are Mean ± SEM of Normal n = 8, FOH n = 4, LOH n = 4, PHH n = 5, SB/HC n = 4. Only PHH showed a significant difference from normal which had undetectable levels so that the difference was effectively from zero protein (*P* < 0.05 indicated by asterisk *). SB/HC also shows an increase but this was not significantly different from normal. Rat (postnatal P1) astrocytes cell lysates were used as a positive control for the GFAP antibody and these are shown in the last lane.

A 53 KDa, GFAP-positive protein band present in the CSF from PHH and SB/HC infants was not detected in normal, FOH and LOH samples (Figure 1a). This GFAP-positive protein was significantly increased in PHH (*P* < 0.05) and showed a non-significant increase in SB/HC CSF when compared with CSF from normal infants (Figure 1b).

### Vimentin

**Figure 2:**
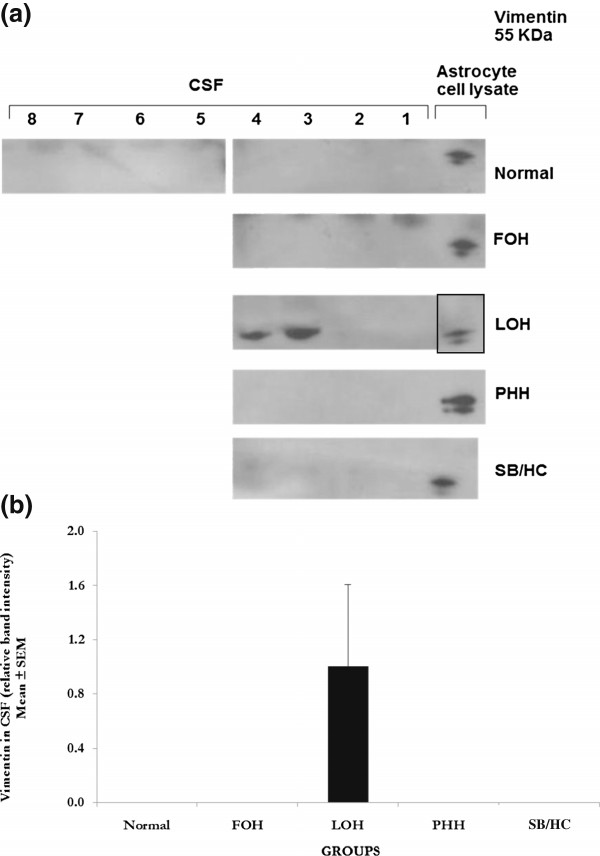
**Western blot analyses for vimentin. a**. **Western blots for vimentin**. Ten-lane precast gels were used. Each lane had an individual 5 μl sample of CSF from the categories of patients shown. After protein separation using SDS PAGE they were blotted onto a PVDF membrane for probing with antibodies. ECL detection was used to visualise the labelled bands and for semi-quantitative analysis using Image-J software. A single 55 KDa band was labelled using the antibody to vimentin and only detected in 2 of the 4 LOH samples or glial cell lysates (last lane except for LOH where it was not run on this particular gel) as shown in these representative gels. All samples were run a minimum of 3 times with a total number of samples as given for semi-quantitative analysis. No significant differences to normal were detected in any group even LOH where two samples had this protein and 2 not. A greater sample number is needed to test if vimentin is generally present in LOH or not. Patient groups as for Figure 1. **b**. **Semi-quantitative analysis** of the 55 KDa band labelled with anti-vimentin antibody relative to the background stain of normal samples where this protein was not detected. Values are Mean ± SEM of Normal n = 8, FOH n = 4, LOH n = 4, PHH n = 4, SB/ HC n = 4. Rat (postnatal P1) astrocyte cell lysates were used as a positive control for the vimentin antibody. No significant differences were found.

A 55 KDa vimentin-positive protein band was observed in the CSF from two infants with LOH but was barely detected in normal, FOH, PHH or SB/HC infants (Figure 2a). The presence or absence of the vimentin positive band in different LOH infants gave a non-significant (*P* > 0.05) statistical test (Figure 2b) against normal. Two of four LOH samples have obviously raised levels of vimentin compared to any other individual tested.

### Myelin basic protein (MBP)

**Figure 3:**
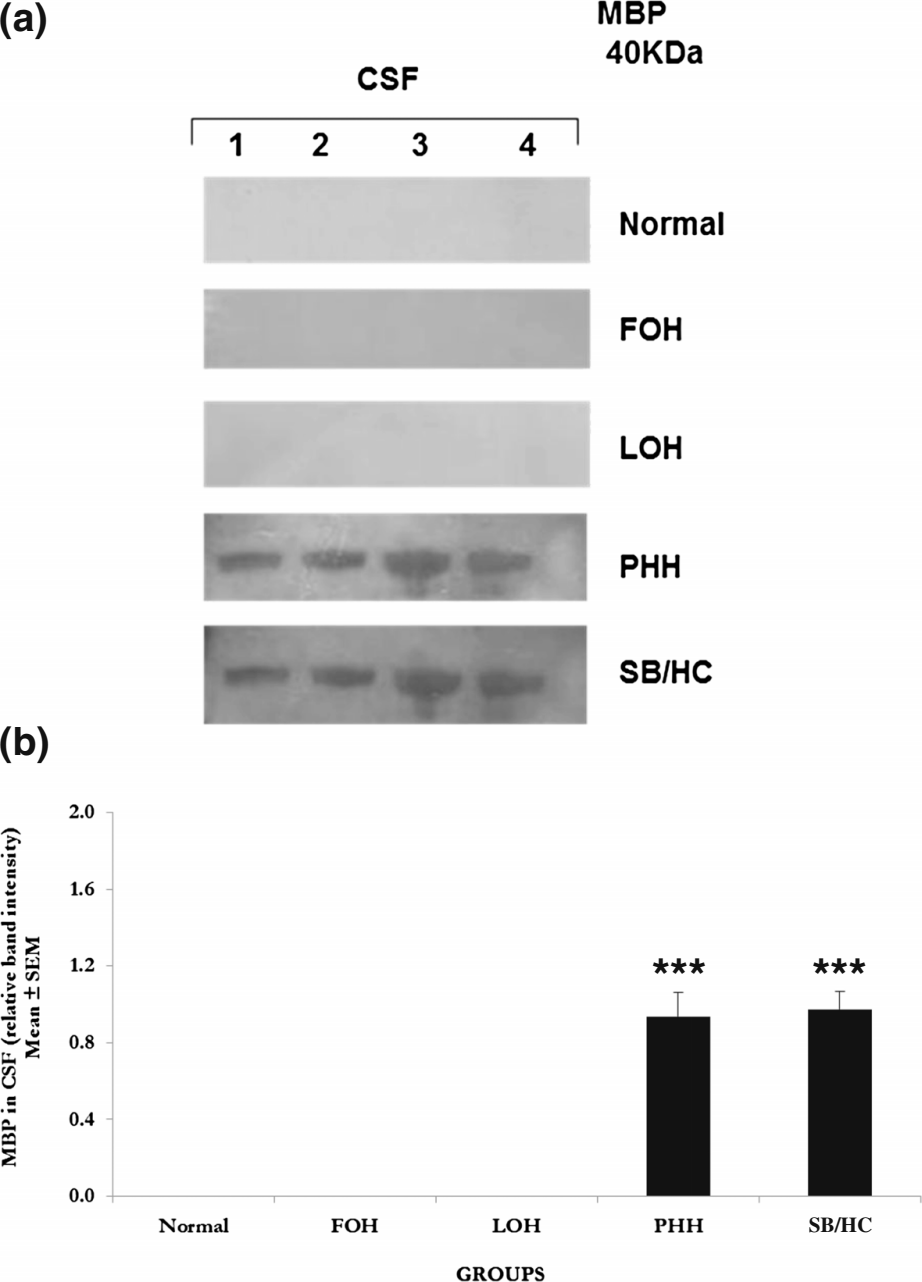
**Western blot analyses for myelin basic protein (MBP). a**. **Western blots for MBP**. Ten-lane precast gels were used. Each lane had an individual 5 μl sample of CSF from the categories of patients shown. After protein separation using SDS PAGE they were blotted onto a PVDF membrane for probing with antibodies. ECL detection was used to visualise the labelled bands and for semi-quantitative analysis using Image-J software. A single 40 KDa band was labelled using the antibody to myelin basic protein and only detected in PHH and SB/HC samples as shown in this representative gel. All samples were run a minimum of three times with a total number of samples as given for semi-quantitative analysis. Patient groups as for Figure 1. **b**. **Semi-quantitative analysis** of the 40 KDa band labelled with anti-MBP antibody. MBP was increased significantly (*P* < 0.001) in PHH and SB/HC compared to undetectable levels in normal, FOH and LOH infants. Values are Mean ± SEM of Normal n = 4, FOH n = 4, LOH n = 4, PHH n = 4, SB/HC n = 4. Values in PHH are SB/HC are significantly different to the undetectable level in normal, so effectively from the zero protein level, *P* < 0.001 indicated by asterisks ***.

A 40 KDa MBP-positive protein band was observed in the CSF from infants with PHH and SB/HC conditions but was below the detection limit in CSF from normal, FOH and LOH infants (Figure 3a). MBP showed a highly significant (*P* > 0.0001) increase in CSF of PHH and SB/HC infants compared to normal. The elevation of MBP in PHH and SB/HC infants showed little variation between different samples analysed.

### 2^′^ , 3^′^-cyclic nucleotide 3^′^-phosphodiesterase (CNPase)

**Figure 4:**
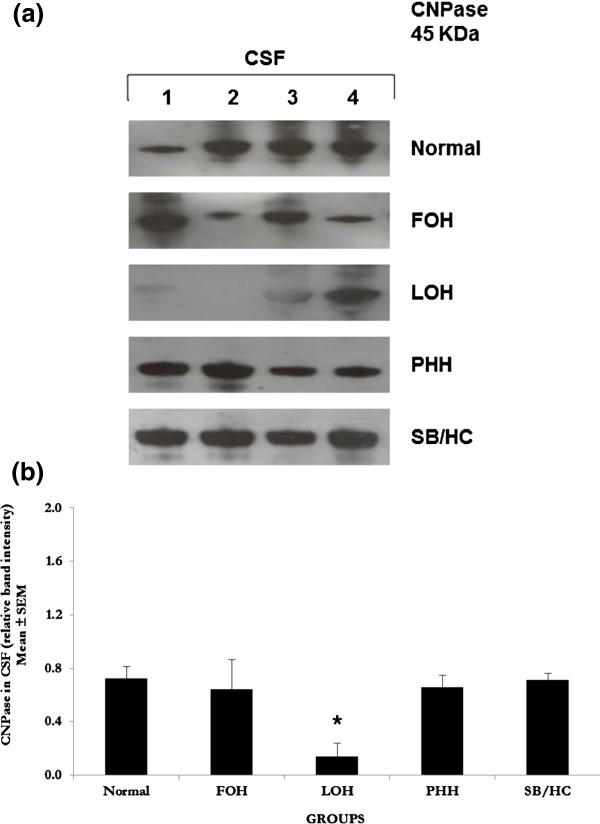
**Western blot analyses for 2**^**′**^** , 3**^**′**^**-cyclic nucleotide 3**^**′**^**-phosphodiesterase (CNPase). a**. **Western blots for CNPase**. Ten-lane precast gels were used. Each lane had an individual 5 μl sample of CSF from the categories of patients shown. After protein separation using SDS PAGE they were blotted onto a PVDF membrane for probing with antibodies. ECL detection was used to visualise the labelled bands and for semi-quantitative analysis using Image-J software. A single 45 KDa band was labelled using the antibody to CNPase and was detected in all samples as shown in these representative gels. All samples were run a minimum of three times with a total number of samples as shown for semi-quantitative analysis. Patient groups as for Figure 1. **b**. **Semi-quantitative analysis** of the 45 KDa band labelled with anti-CNPase antibody shows similar levels of CNPase to that in normal CSF in FOH, PHH and SB/HC but in the LOH group where a significant reduction (*P* < 0.05 indicated by asterisk *) was observed compared to normal and other hydrocephalic groups. In the LOH gel 3 of the 4 samples show reduced CNPase compared to normal and one sample not. Increased numbers will provide better resolution of this particular group. Values are Mean ± SEM of Normal n = 4, FOH n = 4, LOH n = 4, PHH n = 4, SB/HC n = 4.

A 45 KDa CNPase-positive protein band was detected in all samples of CSF analysed (Figure 4a). CNPase was significantly lower in LOH (*P* < 0.05) although one infant had normal levels. CNPase was reduced but without significance in FOH compared with CSF from normal, PHH and SB/HC infants (Figure 4b).

## Discussion

The present study determined CSF levels of GFAP, vimentin, MBP and CNPase in human neonatal hydrocephalus with different aetiologies compared to CSF from neonates with no neurological abnormalities. Although significant differences as well as non-significant indications of differences have been found, the low number of samples available for analysis limited clear conclusions. The study does show that differences are likely to exist and that a larger study is urgently needed. There are clearly sub-categories within each group that show significant changes and so further clinical data may unravel these issues. The proteins we analysed are intermediate filaments found in astrocytes, ependymal cells (GFAP) early glial progenitors and differentiating astrocytes (vimentin) and oligodendrocytes (MBP) and an enzyme associated with the formation of myelin in oligodendrocytes (CNPase). Their presence in CSF is thus thought to indicate cell compromise, breakdown and release of intracellular components. In this study we found that FOH, the congenital form of hydrocephalus, is not associated with any appearance of cell breakdown products in agreement with previous studies demonstrating absence of cell death [36] and decreased cell proliferation with a cell cycle arrest in the H-Tx rat model of FOH [18,19]. LOH had only vimentin detectable in CSF while PHH and SB/HC had GFAP and MBP but not vimentin in CSF. Interestingly, CNPase has never been described in normal/developmental CSF but we found it in all samples of CSF including those from our non-hydrocephalic control infants. We found it reduced in 3 of 4 samples of LOH compared to other samples and it is interesting to speculate whether the 4th sample might have had a non-infectious cause compared to the normal cause in this category of hydrocephalus. Its presence in all neonatal CSF samples analysed suggests a possible role in the immediate post-natal acceleration of gliogenesis. The source of CSF CNPase must surely be oligodendrocytes and their precursors since no other cell has been shown to express this enzyme. This potential role as a secreted molecule remains to be investigated. It would be very interesting to test CNPase on the differentiation of oligodendrocytes from cortical stem/progenitor cells in *in vitro* cell culture as well as to investigate the factors stimulating secretion of this enzyme from oligodendrocytes.

GFAP is an intermediate filament expressed in mature astrocytes [37,38] and immature ependymal cells [39]. Thus the GFAP seen in hydrocephalic CSF may originate from either source as the ependyma is likely to be maturing in the neonatal period. Our findings support those reported for experimental neonatal acquired hydrocephalus [3,4] and also show that congenital and late-onset hydrocephalus, the latter associated with neonatal and postnatal infections, do not present with raised GFAP but that PHH and SB/HC neonates do. GFAP was reported in the CSF of elderly normal pressure hydrocephalus (NPH) patients compared with neurologically healthy age-matched controls [40,41] suggesting a common pathophysiology involving astroglial damage in these types of hydrocephalus, but astroglial protection in FOH and LOH in the neonate. This protection may not be present in older untreated individuals. The latter point is supported by findings in hydrocephalic H-Tx rats in which reactive astrogliosis and microgliosis were observed and correlated with the severity of hydrocephalus and increasing age [42]. Moreover, Del Bigio *et al.* reported significantly increased GFAP levels in hydrocephalic rats which decreased after shunting suggesting an effect of fluid accumulation and/or raised intracranial pressure on these cells [43]. Similar increased GFAP RNA and protein levels were observed in 10-day old kaolin-induced hydrocephalic kittens which also decreased after shunting [44]. Ependymal damage is well documented in hydrocephalus as is astrogliosis so it is perhaps not surprising to find GFAP in CSF as a consequence of specific insults. The finding that GFAP is not present in CSF from FOH and LOH must therefore indicate protection of these cells from damage in these forms of hydrocephalus. Thus, the current study indicates early-stage pathology differs depending on etiology.

MBP in CSF is an indicator of damage specifically associated with myelination as this protein is only known to be present in oligodendrocytes [45,46]. MBP is an important marker to study in hydrocephalus since the degree of pathology and functional neurological deficits are largely associated with lack of, or damage to myelination, e.g. of the corpus callosum and periventricular white matter. We found significantly elevated levels of MBP in the CSF of infants with PHH and SB/HC indicating that demyelination, and/or a failure of myelination is likely to be occurring in these conditions, but not in FOH or LOH. The intraventricular or subarachnoid bleeding in PHH is likely to contribute directly to brain damage [47] and to the demyelination of the periventricular white matter reported in PHH. Sutton *et al.,*[48] reported the presence of MBP protein in the CSF of hydrocephalic patients with different aetiologies and suggested that active hydrocephalus, associated with progressive ventricular dilatation, would produce periventricular demyelination through mechanical stretching of the brain parenchyma. Increased intracranial pressure has a greater impact on early cognitive development than increased CSF volume, and the negative effect is partially reversible through early ventricular shunting [49] giving improved myelination. The fact that MBP is not detected in FOH and LOH again indicates a lack of direct pathology in these specific aetiologies of hydrocephalus and the possibility to prevent loss of myelination through early intervention.

Vimentin is a cytoskeletal intermediate filament involved in maintaining cell integrity in many different cells types including fibroblasts and endothelial cells [50] as well as progenitor cells and early neuronal and glial cells [51-53]. Vimentin is found in neuronal stem and progenitor cells and astrocytes during the early postnatal period and re-expressed in reactive astrocytes in cases of central nervous system injury [54-56]. Microglial cells also express vimentin, specifically after brain damage and/or activation through local inflammatory mediators [57]. In hydrocephalus vimentin-positive cells increase around disrupted areas of ependyma suggesting that reactive microglia and proliferating immature glial cells are associated with areas of ependymal cell loss [58]. Our data would further suggest that these cells may be compromised by fluid accumulation and raised intracranial pressure. The increased levels of vimentin in the LOH infants must be a direct consequence of infection affecting the brain but the lack of GFAP in LOH indicates that the source is unlikely to be astroglial and may be microglial, endothelial or leptomeningeal since many patients present with meningitis. This requires further analysis as it indicates a very different pathophysiology in these patients compared to those with other aetiologies of hydrocephalus.

CNPase is a protein accounting for approximately 4% of myelin protein content and is considered an index of myelin formation [59,60] where the amount of immunoreactive CNPase correlates with the thickness of the myelin sheath in the central nervous system [61,62]. Wu and colleagues [63] reported that they also found CNPase expressed in prenatal and early postnatal microglial cells in rat brain with a gradual decrease with age, essentially undetectable after birth. They suggested that downregulation of CNPase related to the transformation of microglia from the mobile and amoeboid type to the ramified type during development. If confirmed the finding of CNPase in non-myelin producing cells presents a further mystery regarding its functions along with its presence in CSF found in the current study. Absence of CNPase results in serious conditions. CNPase-knockout mice show severe symptoms, including convulsions and ataxia, and most homozygous mice die between 6 and 12 months of age [64]. Decrease in CNPase expression is also observed in chronic schizophrenic patients [65] indicating a possible involvement in the underlying developmental pathology. In kaolin-induced hydrocephalic rats Del Bigio *et al.*[43] reported decreased CNPase in the corpus callosum/supraventricular white matter, fimbria, medulla, and spinal cord indicating a sensitivity of myelin in these regions to raised intracranial pressure. Furthermore, a reduction in the number of CNPase immunoreactive oligodendrocytes both in the subependymal layer and the cerebral cortex of hydrocephalic rat brains correlated with the severity of the hydrocephalus [66]. CNPase has not been described in neonatal CSF before this study and was an unexpected finding particularly its presence in all neonatal CSF samples tested. LOH had reduced CNPase in CSF compared to normal and other conditions. As CNPase is thought to be involved in oligodendrocyte surface membrane expansion and migration during early stages of axonal ensheathment [67], its presence in CSF may indicate a potential role in global brain myelination not previously considered. This requires further study but may prove to be an important tool in promoting myelination in affected brains.

This study has a number of limitations. Firstly, to obtain CSF free of contamination excluded many collected samples so that a much larger study is required to investigate our current findings in more detail. Secondly, it is practically impossible to obtain CSF samples through ventricular catheterisation from healthy neonates due to technical limitations and ethical issues. Similarly, it is unusual to obtain lumbar CSF from hydrocephalic patients. Although some reports indicate that ventricular CSF may be more stable and accurate for measuring pathological changes [68], others contradict this [69]. For our study, all normal samples were taken by lumbar puncture while all hydrocephalic CSF came from lateral ventricles. We assumed that only obstructive hydrocephalus would produce a change in ventricular CSF that was not reflected in lumbar drainage but also acknowledge reported differences in total protein between lumbar and ventricular CSF which we believe would not affect the validity of our findings since we are looking for changes in specific proteins associated with pathology. Of note here is the finding of CNPase in both normal and affected neonatal CSF at equal levels (except in LOH) that supports an argument that the measurements are comparable in this study.

Taken together, the findings of this study indicate that different aetiologies leading to hydrocephalus are associated with different pathophysiological mechanisms affecting different cell types, at least in the initial stages where intracranial pressure may not be pathologically raised. The congenital form of hydrocephalus has no detectable pathological markers in CSF but is known to be affected by a physiological block of cell cycle as well as a folate block [18,19]. Differential diagnosis is therefore possible and the potential for more effective treatment of these different conditions may emerge from further research. Further studies are clearly needed and, with more detailed clinical data, e.g., measurements of ventriculomegally and additional radiographical data on brain measures, developmental and neurological signs and symptoms, as well as pre- and post-surgical data, a more robust differential diagnostic is likely to emerge which would feed into research for differential treatment depending on the characterisation of the underlying pathology. Furthermore, we have focused on glial cell effects but additional data on neuronal status is also needed in differentiating between the different aetiologies leading to hydrocephalus.

## Conclusions

This study has demonstrates that different aetiologies leading to neonatal hydrocephalus can likely be differentiated by CSF analysis. This presents an important step in understanding this devastating neurological condition. These results suggest that early-stage changes are associated, not with raised pressure, but with currently unknown physiological changes that affect different cell types in different locations within the hydrocephalic brain. Shunting will only address the common end point of these different aetiologies and is unlikely to address the underlying differences in physiology. It is therefore important to understand these physiological processes in order to prevent irreversible cell damage and lasting neurological deficits.

## Abbreviations

CNPase: 2′, 3′-cyclic nucleotide 3′-phosphodiesteras; CSF: Cerebrospinal fluid; FOH: Fetal-onset hydrocephalus; GFAP: Glial fibrillary Acidic protein; H-Tx: Hydrocephalic Texas rat; LOH: Late onset hydrocephalus; MBP: Myelin basic protein; PHH: Post-haemorrhagic hydrocephalus; SB/HC: Spina bifida with hydrocephalus.

## Competing interests

The authors declare they have no competing interests.

## Authors’ contributions

JAM and IN conceived and designed the study. IN carried out all of the lab work. KAIW, AWR, SV, CM and JRG were involved in providing clinical samples, discussing the study design and data collected. SNC coordinated the work in Pakistan. All authors contributed to various drafts of the manuscript and have read and approved the final version.
